# Rational Drug Use Support System: design, artificial intelligence integration, and pilot feasibility trial

**DOI:** 10.1590/1806-9282.20260803

**Published:** 2026-10-16

**Authors:** Mustafa Kahraman, Meris Esra Bozkurt, Soner Kotan, Buse Nur Ozturk, Rana Aydın, Saim Hatipoğlu, Hasan Ali Eris, Mehmet Ali Erturk, Muhammed Ali Aydin, Mehmet Akif Karan

**Affiliations:** 1 Türkiye Public Health and Chronic Diseases Institute – Istanbul, Türkiye.; 2 Mersin University, Mersin Medical School, Department of Internal Medicine, Division of Geriatrics – Mersin, Türkiye.; 3 Türkiye Health Data Research and Artificial Intelligence Applications Institute – Istanbul, Türkiye.; 4 Istanbul University, Istanbul Faculty of Medicine – Istanbul, Türkiye.; 5 Istanbul Gelisim University, Faculty of Engineering and Architecture, Department of Software Engineering – Istanbul, Türkiye.; 6 Istanbul University-Cerrahpasa, Department of Computer Engineering – Istanbul, Türkiye.; 7 Istanbul University, Department of Computer Engineering – Istanbul, Türkiye.; 8 Istanbul University, Istanbul Faculty of Medicine, Department of Internal Medicine, Division of Geriatrics – Istanbul, Türkiye.

**Keywords:** Digital health, Drug adherence, Telemedicine, Artificial intelligence, Mobile health

## Abstract

**OBJECTIVE:**

The Rational Drug Use Support System was developed to assist patients in using the right drug, at the right time and at the right dose according to their treatment protocols. The aim of this study was to evaluate the performance of an artificial intelligence-enhanced mobile application designed to support rational drug use and to assess its feasibility and usability through a pilot trial.

**METHODS:**

The system includes an artificial intelligence -powered mobile application and a web-based interface integrating convolutional neural networks and object detection algorithms for human activity recognition. To build an image library, real patient videos were recorded in a hospital setting under physician supervision. A total of 357 original medication-intake videos were collected from 31 patients, which were then segmented into 1,626 short clips to train the artificial intelligence model. The system was first tested under controlled laboratory conditions and then evaluated for feasibility in a hospital pilot trial involving eight participants over 15 days.

**RESULTS:**

The proposed artificial intelligence model achieved 94% accuracy in recognizing medication intake activities in the test set, suggesting promising performance that warrants further evaluation. Exploratory analyses of the 36-Item Short Form Health Survey Questionnaire administered to patients who utilized the mobile application for 15 days revealed improvements in the domains of social functioning, vitality, and mental health.

**CONCLUSION:**

The findings suggest that the "Rational Drug Use Support System" appears feasible for implementation in telemedicine and clinical settings to support rational drug use and patient engagement in chronic disease management. However, larger controlled studies are warranted to confirm these preliminary findings.

## INTRODUCTION

Chronic diseases remain a major global health challenge and are responsible for a substantial share of mortality, years of life lost, and long-term morbidity worldwide^1,2^. A major barrier to effective chronic disease management is rational drug use and deviation from prescribed treatment protocols. The World Health Organization has long emphasized that medication-related problems are common and that many patients do not take medicines as prescribed^3^. Poor adherence can reduce treatment effectiveness, increase preventable complications and hospitalizations, and, particularly in antimicrobial use, contribute to drug resistance. In practice, inappropriate drug use may include missed doses, delays, or self-directed changes in dose or treatment duration, all of which can lead to substantial clinical and economic consequences.

Digital health interventions, particularly mobile health (mHealth) applications, have therefore gained attention as scalable tools to support self-management, especially in settings where smartphone use is widespread^4^. In this context, digitally supported care models such as telehealth and telemedicine have become increasingly relevant for maintaining continuity of care, supporting rational drug use, and reducing avoidable healthcare use^5,6^.

In this study, we developed an artificialintelligence (AI)-driven Rational Drug Use Support System to support patients in taking the right medication at the right time and dose. The system was developed and evaluated in a real hospital setting under clinical supervision. The study aimed to assess the performance of the AI-enabled system in objectively verifying medication intake activities and to evaluate its feasibility and usability in routine clinical practice through a pilot trial.

## METHODS

### Study design and participants

This study used a multi-stage design to develop and clinically evaluate a Rational Drug Use Support System. The clinical component was designed as a feasibility pilot study rather than an efficacy trial. A preliminary survey of 300 patients was conducted to examine factors related to rational drug use and technology use among individuals with chronic diseases^7^. Of these, 31 volunteered to contribute to image library development, and eight were subsequently selected by computer-generated randomization for inclusion in the hospital-based pilot trial. Eligible participants were adults aged 18 years or older who were receiving continuous medication, able to swallow and self-administer medication independently, capable of providing written informed consent, and had basic technological literacy. Individuals with cognitive impairment, severe sensory or functional limitations affecting independent drug use, severe visual impairment, life expectancy under two years, pregnancy or breastfeeding, or inability to self-administer medication were excluded.

### Image library development and artificial intelligence model training

Video data were collected in the inpatient unit of a university internal medicine department, under physician supervision and in accordance with privacy safeguards. Before recording, demographic characteristics, prescribed medication count, and general health status were documented, and body mass index (BMI) was calculated as weight in kilograms divided by height in meters squared^8^. To increase variability in the training data, participants were recorded while sitting, standing, and lying in bed. Videos were captured using three smartphones mounted on tripods and positioned to provide a clear view of the face and upper body. Each recording followed a two-step procedure in which the participant first showed the medication package or blister pack to the camera and then completed the medication-taking action. For each participant, 10–14 recordings were obtained under varying hand-use, lighting, and background conditions. Individual recordings lasted 2–6 s, yielding 357 original videos from 31 participants. All videos were anonymized and securely transferred for AI model development.

For model development, the 357 original recordings were randomly partitioned into training, validation, and test sets at a ratio of 60:20:20. The training set was used for model parameter optimization, the validation set was used for hyperparameter tuning and early stopping to prevent overfitting, and the test set was reserved exclusively for final performance evaluation.

From these 357 recordings, 1,626 short clips of 2–6 s were generated to capture different stages of medication intake and increase within-class variability. To improve generalizability and reduce confusion with visually similar actions, the dataset was expanded with additional eating clips from publicly available sources and the UCF101 (A Dataset of 101 Human Action Classes From Videos in the Wild) Human Activity Recognition dataset, together with clips from 11 unrelated activities used as negative examples.

Medication-intake actions were detected using the Personalized Human Activity Recognition (PHAR) framework^9^, which combines object detection with human activity recognition (HAR) to focus on regions most relevant to the target behavior. Two PHAR-based architectures were implemented in line with previous HAR studies: a Convolutional Neural Network Long Short-Term Memory (CNN–LSTM) model and a 3D convolutional neural network (3D-CNN)^7,,9,10,11^. These were developed as two model variants, Two-Dimensional Motion Feature Maps (MFM2D) and Three-Dimensional Motion Feature Maps (MFM3D), which shared the same input structure and preprocessing pipeline but differed in temporal modelling.

The PHAR input consisted of 16 temporally ordered frames, referred to as the Main Frame sequence (MFRM). Because medication-taking and drinking actions mainly involve coordinated movements of the face, hands, and upper body, object detection was used to generate three additional focus views for each frame: Face Frame (FFRM), Hands Frame (HFRM), and Person Frame (PFRM).

The validation set was used to monitor training and limit overfitting, whereas the test set was reserved for final performance evaluation. Because the dataset contained a substantial number of negative examples, model performance was assessed not only by accuracy but also by precision, recall, and F1-score for the three target classes: Taking Medicine, Drinking Water, and Eating. The F1-score was considered the primary balanced performance metric.

### System architecture and functionality

The Rational Drug Use Support System was implemented as a client–server architecture comprising a physician-facing web platform, a patient-facing mobile application, and a central server hosting the trained PHAR model. After authentication, it synchronized the patient's medication schedule, delivered reminders at predefined times, and prompted the user to record a short video of the medication intake process. These data were securely transmitted to the server, where the PHAR model classified the action and generated a medication intake outcome.

### Pilot feasibility study

Feasibility evaluation was conducted through a prospective observational pilot study involving eight participants selected from the video dataset group. The study period was 15 days. The primary focus of this pilot was to assess feasibility, usability, and user acceptance rather than clinical efficacy. Key feasibility outcomes included successful video uploads, system usability, application adherence, and medication verification success rates. At baseline, all participants received standardized training on application use and alert messages. Physicians created user accounts through the web platform and entered the prescribed treatment schedules, which were then synchronized to participants’ smartphones. Secondary exploratory outcomes were assessed using the 36-Item Short Form Health Survey (SF-36) Short Form Health Survey at baseline (day 0) and post-intervention (day 15), together with a study-specific patient satisfaction questionnaire^12–14^.

### Statistical analysis

All data were recorded and analyzed using Statistical Package for the Social Sciences (SPSS) Statistics (version 25.0; IBM/SPSS). Analyses were performed separately for the video dataset group and the clinical pilot group. Normality of continuous variables was assessed using the Kolmogorov-Smirnov test. Normally distributed variables are presented as mean±standard deviation, and non-normally distributed variables as median (min–max). Associations between categorical variables were examined using the chi-square test. For the pilot study, SF-36 scores at baseline and day 15 were compared using paired analyses, with the paired t-test applied for normally distributed differences and the Wilcoxon signed-rank test used otherwise. Statistical significance was set at a two-sided p<0.05.

## RESULTS

### Participant characteristics

The image library cohort comprised 31 participants with a mean age of 44±18 years, of whom 67.7% were female. Polypharmacy, defined as the use of four or more medications, was observed in 6.5% of participants. Demographic and clinical characteristics of this cohort are presented in Table 1.

**Table 1:** Basic data of participants who used to mobile application by gender.

		Male (n=3) 37. 5%	Female (n=5) 62.5%	Total (n=8) 100%	p-value
Age (year)*		23 (20–53)	20 (20–49)	28 (20–53)	0.341
BMI (kg/m^2^)		28.8+0.9	22.9+1.7	25.1+3.3	0.002Ø
Marital status (n, %)					
	Married	1 (33.3%)	1 (20.0%)	2 (25.0%)	0.673
	Single	2 (66.7%)	5 (80.0%)	6 (75.0%)	
Living situation (n, %)					
	Family	0 (0.0%)	2 (40.0%)	5 (62.5%)	0.028Ø
	Alone	3 (100%)	3 (60.0%)	3 (37.5%)	
Education (n, %)					
	High school	0 (0.0%)	1 (20.0%)	1 (12.5%)	0.408
	College-faculty	3 (100%)	4 (80.0%)	6 (87.5%)	
Profession (n, %)					
	Housewife	0 (0.0%)	1 (20.0%)	6 (75.0%)	0.315
	Studen t	2 (66.7%)	4 (80.0%)	1 (12.5%)	
	Teacher	1 (33.3%)	0 (0.0%)	1 (12.5%)	
Income level (n, %)					
	Minimum wage	0 (0%)	0 (0%)	1 (12.5%)	0.315
	Between minimum wage and twice	2 (66.7%)	1 (20%)	6 (75.0%)	
	Minimum wage is between 2 and 4 times	1 (33.3%)	4 (80%)	1 (12.5%)	
	Chronic medication using status (n, %)	2 (66.7%)	1 (20%)	3 (37.5%)	0.187
Time to take medication					
	Morning	1 (33.3%)	4 (80.0%)	5 (62.5%)	0.293
	Evening	1 (33.3%)	0 (0.0%)	1 (12.5%)	
	Noon	1 (33.3%)	1 (20.0%)	2 (25.0%)	
	The state of forgetting the medication time (n, %)	2 (66.7%)	3 (60.0%)	5 (62.5%)	0.850
Frequency of forgetting the medication time (n, %)					
	Often	2 (66.7%)	2 (40.0%)	4 (50.0%)	0.641
	Sometimes	0 (0.0%)	1 (20.0%)	1 (12.5%)	
	Never	1 (33. 3%)	2 (40.0%)	3 (37.5%)	
The state of forgetting the medication according to the hungry or full state (n, %)					
	Often	0 (0.0%)	1 (20.0%)	1 (12.5%)	0.408
	Never	3 (100%)	4 (80.0%)	0 (0.0%)	
	Presence of situation of discarding medication because it has expired (n, %)	3 (100%)	4 (80.0%)	7 (87.5%)	0.408
Number of boxes of drugs discarded because the expiration date has passed (n, %)					
	0 box	0 (0.0%)	1 (20.0%)	0 (0.0%)	0.408
	1–3 boxes	3 (100%)	4 (80.0%)	7 (87.5%)	
	4–7 boxes	0 (0.0%)	0 (0.0%)	0 (0.0%)	
	Presence of unfinished medication in home (n, %)	3 (100%)	4 (80%)	7 (87.5%)	0.408
Number of unfinished medicine boxes at home (n, %)					
	0 box	0 (0.0%)	1 (20.0%)	0 (0.0%)	0.376
	1–3 boxes	1 (33.3%)	3 (60.0%)	4 (50.0%)	
	4–7 boxes	2 (66.7%)	1 (20.0%)	3 (37.5%)	
	Situation of getting help with medication using (n, %)	1 (33.3%)	1 (20.0%)	2 (25.0%)	0.673
	Knowing the "e- nabiz" application (n, %)	2 (66.7%)	5 (100%)	7 (87.5%)	0.168
Opinion about the "e-nabiz" application (n, %)					
	Usefu l	2 (66.7%)	4 (80.0%)	6 (75.0%)	0.673
	Undecided	1 (33.3%)	1 (20.0%)	2 (25.0%)	
Opinion about the rational drug use support system mobile application (n, %)					
	Useful	1 (33.3%)	5 (100%)	6 (75.0%)	0.035Ø
	Undecided	2 (66.7%)	0 (0.0%)	2 (25.0%)	

m: meter; kg: kilogram; BMI: body mass index. Data are given as mean±standard deviation, median (interquartile range) or number (percentage) as applicable.

* Given data as median (interquartile range).

Ø Significant p-values.

The pilot study included eight participants with a mean age of 38 years (range: 30–53) and a mean BMI of 25.3±3.3 kg/m². Several indicators pointed to a need for medication intake support: 50% reported frequently forgetting medication intake, 25% reported receiving assistance for drug use, and 87.5% reported both discarding 1–3 boxes of medication annually due to expiry and keeping unfinished medication boxes at home. Before using the system, 75% indicated that they expected the application to be beneficial. Participant characteristics for the pilot group are summarized in Table 2. Within this small sample, gender differences were observed for BMI (p=0.002) and living situation (p=0.028).

**Table 2:** Comparison of the general quality of life of the participants using the mobile application, evaluated by the 36-item Short Form Health Survey scale before and after the application using.

	SF-36 scale before using application	SF-36 scale after using application	p-value
1. Physical functioning (%)	93.7±11.5	93.7±9.1	1
2. Role limitations due to physical health (%)	84.3±29.6	90.6±26.5	0.32
3. Role limitations due to emotional problems (%)	29.1±33.3	54.1±50.2	0.08
4. Energy/fatigue (%)	44.3±24.9	51.2±22.3	0.001*
5. Emotional well-being (%)	61.0±18.9	67.0±16.2	0.03*
6. Social functioning (%)	78.1±24.7	81.2±25.0	0.02*
7. Pain (%)	83.7±15.6	80.9±16.3	0.46
8. General health (%)	70.6±17.4	71.8±12.5	0.10

SF-36: The 36-item Short Form Health Survey. Data are given as mean±standard deviation, median (interquartile range) or number (percentage) as applicable.

‘*’ The symbol represents significant p-values.

### Artificial intelligence model performance

The proposed AI model achieved 94% accuracy on the test set in recognizing medication intake activities. The MFM3D variant demonstrated superior performance compared to the MFM2D variant, particularly for the "Taking Medicine" activity class. Precision, recall, and F1-scores for the target classes were consistently high (>0.90), indicating robust classification performance.

### Pilot feasibility outcomes

With regard to feasibility outcomes, all eight participants successfully completed the 15-day study period. The application adherence rate was high, with participants recording videos for the majority of their scheduled medication intake times. Successful video upload rates exceeded 90%, and the system demonstrated reliable medication verification. System usability was rated favorably by participants.

### Exploratory health-related quality of life outcomes

Health-related quality of life was assessed using the SF-36 at baseline and after 15 days of application use. General health scores did not change significantly (70.6±17.4 vs. 71.8±12.5; p=0.104). Exploratory analyses revealed statistically significant improvements in vitality/energy (p=0.001), mental health/emotional well-being (p=0.031), and social functioning (p=0.019), whereas no significant differences were found in the remaining domains (Table 2).

### User satisfaction

At the end of the pilot, participants reported high satisfaction with the system. Overall, 75% stated that the application supported rational drug use of their medication regimen, while 87.5% reported that reminders were effective and that the system helped them manage drug use independently. Interoperability was also identified as a key priority, with 87.5% indicating that integration with public and private hospitals, pharmacies, and the national e-Nabiz ^®^ personal health system would be useful (Table 3).

**Table 3:** Satisfaction survey of users of the Rational Drug Use Support System mobile application regarding the pilot trial.

	Opinion useful group	Opinion non useful group
Did the mobile application help you comply by the medication treatment protocol?	6 (75.0%)	2 (25.0%)
Did the mobile application successfully perform your medication time reminders?	7 (87.5%)	1 (12.5%)
Did the mobile application successfully perform fasting/satiety reminders for medication use?	6 (75.0%)	2 (25.0%)
Were you able to use your medicines without any help from anyone with the support of the mobile application?	7 (87.5%)	1 (12.5%)
Did your medication using regulated in your daily life thanks to the use of the mobile application?	6 (75.0%)	2 (25.0%)
Are you satisfied with using the mobile app?	6 (75.0%)	2 (25.0%)
Would you like the mobile application to work integrated with all public/private hospitals?	7 (87.5%)	1 (12.5%)

## DISCUSSION

This study describes the development and preliminary feasibility evaluation of an AI-enabled Rational Drug Use Support System that integrates a PHAR pipeline for the objective verification of medication intake. The primary aim was to assess the performance of the AI system and evaluate its feasibility and usability in a clinical setting, rather than to establish clinical effectiveness. Two main findings emerged. First, the proposed PHAR variants (MFM2D and MFM3D) outperformed the baseline architectures used in this study, particularly for the "Taking Medicine" activity. Second, in the short hospital-based pilot, participants demonstrated high engagement with the system and reported favorable usability, together with exploratory improvements in selected SF-36 domains.

Previous studies on medication intake monitoring have reported a wide range of classification performance across image- and sensor-based systems. Scoping evidence suggests that accuracies often remain below the mid-90% range^15^, although some multimodal approaches have achieved higher values under specific conditions^16^. The performance observed in the present study is broadly consistent with this literature and supports the feasibility of vision-based approaches for objective intake verification in controlled settings. Importantly, reducing false positives is likely to be critical for sustained use, as frequent incorrect alerts or non-adherence flags may increase user burden and contribute to disengagement over time^17^. From this perspective, combining personalized attention to relevant body regions with temporal modelling may improve the reliability of AI-driven monitoring, particularly when activities are visually confusable.

Although the pilot was not designed to establish clinical efficacy, participant-reported outcomes showed exploratory improvements over the 15-day period in vitality, mental health, and social functioning. The direction of change observed in this pilot is also consistent with broader evidence suggesting that mHealth interventions can support self-management and may improve patient-reported outcomes in chronic illness^18^. However, given the exploratory design of the study, the absence of a control group, and the small sample size, these findings should be interpreted as preliminary and hypothesis-generating rather than as evidence of clinical effectiveness. It is possible that the observed improvements reflect regression to the mean, placebo effects, or increased attention from study participation rather than the intervention itself. These findings require confirmation in larger and more rigorous evaluations with control groups and longer follow-up periods.

User feedback also supported the feasibility of the system. Most participants reported that reminders were helpful and that the system enabled them to manage drug use more independently, in line with earlier findings on AI-assisted medication management tools^19^. Participants also emphasized the importance of interoperability, particularly integration with hospitals, pharmacies, and the national personal health record platform, e-Nabiz ^®20^. Such integration could simplify medication scheduling, reduce manual data entry, and support continuity across care settings, although it would also require careful attention to governance, authentication, and data protection.

A major strength of this study is the end-to-end development and evaluation of an integrated medication intake support system using real patient videos collected in a clinical environment. This setting enhances the ecological validity of both the model-development pipeline and the usability assessment. In addition, combining patient-reported Health-Related Quality of Life (HRQoL) (SF-36) with satisfaction feedback provides a broader perspective on both experiential and outcome-related dimensions.

Several limitations should be acknowledged. The pilot included a small sample (n=8) and a short follow-up period of 15 days, which limits generalizability and precludes firm conclusions about clinical effectiveness. The absence of a control group further limits the interpretation of the observed SF-36 changes. In addition, the study population was relatively young, and the proportion of participants with polypharmacy was low, which may limit the applicability of the findings to older adults and patients with cognitive impairment who are at highest risk for medication-related problems. The HRQoL changes observed should therefore be regarded as exploratory and hypothesis-generating.

Future research should include larger, controlled trials with longer follow-up periods to evaluate the clinical effectiveness of the system. Studies should also specifically target older adult populations and patients with polypharmacy, who stand to benefit most from such interventions. Additionally, objective measures of medication adherence should be incorporated alongside patient-reported outcomes to provide a more comprehensive assessment of the system's impact.

## CONCLUSION

This study introduced and preliminarily evaluated an AI-enabled Rational Drug Use Support System designed to support rational drug use via automated activity recognition within a mobile, patient-facing workflow. The findings suggest that the system appears feasible for implementation in clinical settings, with high AI classification accuracy and favorable user satisfaction reported in a small pilot sample. However, the observed improvements in HRQoL domains should be interpreted as exploratory, and larger controlled studies are needed to confirm these preliminary findings and establish clinical effectiveness. Overall, the findings support the feasibility of combining mobile health delivery with AI-based verification as a complementary approach to conventional chronic disease management.

## Data Availability

The datasets generated and/or analyzed during the current study are available from the corresponding author upon reasonable request.
